# Autism Screening in Early Childhood: Discriminating Autism From Other Developmental Concerns

**DOI:** 10.3389/fneur.2020.594381

**Published:** 2020-12-10

**Authors:** Neil Brewer, Robyn L. Young, Carmen A. Lucas

**Affiliations:** College of Education, Psychology & Social Work, Flinders University, Adelaide, SA, Australia

**Keywords:** autism screening, early childhood, level 2 screeners, STAT, biscuit, ADEC, SORF, RITA-T

## Abstract

Early identification of autism, followed by appropriate intervention, has the potential to improve outcomes for autistic individuals. Numerous screening instruments have been developed for children under 3 years of age. Level 1 screeners are used in large-scale screening to detect at-risk children in the general population; Level 2 screeners are concerned with distinguishing children with signs of autism from those with other developmental problems. The focus here is evaluation of Level 2 screeners. However, given the contributions of Level 1 screeners and the necessity to understand how they might interface with Level 2 screeners, we briefly review Level 1 screeners and consider instrument characteristics and system variables that may constrain their effectiveness. The examination of Level 2 screeners focuses on five instruments associated with published evaluations in peer-reviewed journals. Key criteria encompass the traditional indices of test integrity such as test reliability (inter-rater, test-retest) and construct validity, including concurrent and predictive validity, sensitivity (SE), and specificity (SP). These evaluations reveal limitations, including inadequate sample sizes, reliability issues, and limited involvement of independent researchers. Also lacking are comparative test evaluations under standardized conditions, hindering interpretation of differences in discriminative performance across instruments. Practical considerations constraining the use of such instruments—such as the requirements for training in test administration and test administration time—are canvassed. Published Level 2 screener short forms are reviewed and, as a consequence of that evaluation, future directions for assessing the discriminative capacity of items and measures are suggested. Suggested priorities for future research include targeting large and diverse samples to permit robust appraisals of Level 2 items and scales across the 12–36 month age range, a greater focus on precise operationalization of items and response coding to enhance reliability, ongoing exploration of potentially discriminating items at the younger end of the targeted age range, and trying to unravel the complexities of developmental trajectories in autistic infants. Finally, we emphasize the importance of understanding how screening efficacy is dependent on clinicians' and researchers' ability not only to develop screening tests but also to negotiate the complex organizational systems within which screening procedures must be implemented.

## Introduction

One focus for autism researchers from various disciplinary backgrounds (e.g., genetics, neuroscience, psychiatry, psychology, virology) has been the identification of markers of the condition that can be detected reliably in infancy and early childhood, and incorporated into screening measures for identifying children showing early signs. For many reasons (1), this is a difficult task. Yet, researchers have persisted, motivated perhaps by two considerations. First, it is widely accepted that the optimum developmental outcomes for individuals with autism will be facilitated by implementing appropriate intervention strategies from an early age (2, 3). Second, established and comprehensive diagnostic instruments such as the Autism Diagnostic Observation Schedule (ADOS) (4), the Autism Diagnostic Observation Schedule—Toddler Module (ADOS-T) (5) which is designed specifically for children younger than 30 months, and the Autism Diagnostic Interview-Revised (ADI-R) (6) are not practical for screening purposes due to their lengthy administration time and demanding administrator training. It is important to stress, of course, that comprehensive assessment using such instruments should be conducted when screening measures indicate the possibility of an autism diagnosis, thereby enabling clinicians to confirm any diagnosis and identify any co-occurring conditions that may underlie any symptoms thought to be associated with autism.

Researchers' efforts have now resulted in various screening devices that focus on behavioral markers of autism and specifically target its early detection. Screening instruments for very young children fall into one of two categories: Level 1 screeners used primarily in large-scale screening to identify children in the general population whose developmental trajectory may indicate autism, and Level 2 screeners designed specifically to differentiate young children with autism from those with other developmental concerns. Here we first outline criteria for evaluating these instruments. The behaviors targeted and the rationale for their inclusion are then examined. After a brief review of Level 1 screeners which is important for understanding their relationship to Level 2 screeners, we provide a detailed evaluation of Level 2 screeners. We highlight some practical issues with these measures and examine recently developed short forms, their development motivated by the desire to offer more congenial instruments for practitioners. In so doing, we highlight the potential for misleading conclusions that can arise regarding the discriminative capacity of different instruments. We conclude by identifying major priorities for future research.

## Criteria for Evaluating ASD Screeners

An initial consideration is the source of the screening information. Some screeners rely upon the reports of parents or caregivers about the child's behavior, reports that might be obtained via an interview or checklist completion. Others involve observation of the child's behavior as they engage in a live (or video recorded) interaction with the parent and/or examiner. Both approaches have limitations. For example, parents may not be sensitive to the occurrence, deviation or absence of particular behaviors that may point to developmental concerns (7, 8) or, in response to their child's functioning, may have developed compensatory strategies to minimize perceived deviation or to involve their children in various social interactions (9). If the screening relies upon parents' retrospective reports, other issues may arise should the parent not accurately recall certain behavioral patterns or when they were detected. Observational measures are often labor-intensive and may be insensitive to the behaviors of interest because they only capture a limited behavioral sample due to the duration of the testing session, the responsiveness of the child at the time of observation or the dynamics of the interaction between the child and the parent or clinician. A tightly controlled direct comparison of these approaches with the same children is extremely difficult given the difficulties associated with producing comparable operationalizations of items for each approach. Given the potential biases that may influence parental reports, researchers have generally argued for the superiority of observational measures, although some research runs counter to that view. Moreover, under some conditions, parents may observe clinically significant behaviors sooner than clinicians (10, 11). Note, however, that in these studies, parents were reporting behaviors of younger siblings of an older child with autism and, thus, may have been sensitized to the behaviors.

Another consideration relates to the age and developmental levels of the children sampled in the construction and evaluation of the screener. For example, the effectiveness of screening children below 3 years of age, when using instruments evaluated with children beyond 3 years, is obviously unknown. And, even if the screener were developed using samples of very young children, it is important to establish that the specific behavioral patterns targeted as indicators of potential developmental concerns have some longer term predictive validity of an autism diagnosis, rather than simply being atypical behaviors that are still within the boundaries of a typical developmental trajectory. The developmental levels of children sampled are also important. For example, instruments evaluated using samples from clinics accessing a preponderance of children with relatively severe developmental concerns may have shown strong discriminative performance and yet not be so effective at discriminating children with more subtle symptomatology or autism specifically.

The practicalities of screening are also important. That there may be practical constraints on screening implementation is indicated by findings that, despite recommendations from organizations such as the American Academy of Pediatrics that all children be screened at 18 and 24 months (12), reported rates of screening of children in this age range among pediatricians and physicians vary widely—for example, from 22% (13) to more than 85% (14). Some obvious constraints on screening are the administration time, lack of familiarity with and training in both the administration and scoring of the screener, and system variables associated with making post-screening referrals and following up outcomes (15, 16).

A fourth criterion is the instrument's reliability. Inter-rater reliability and test-retest reliability are important considerations when evaluating any psychometric instrument, but they warrant emphasis in this context for two reasons. First, very young children are likely to show considerable intra-individual variability in reactivity or responsiveness during interpersonal interactions. Second, as will become apparent when we discuss the specific types of behaviors that these instruments are trying to assess, there is considerable potential for individual observers—whether they be parents or experienced clinicians—to vary in their evaluations of whether the presence, or absence, of certain behaviors meet criterion for passing or failing a test item.

The issue of how effectively the instrument is measuring the construct of interest is an important fifth criterion. The items should converge on the focal construct(s) or dimension(s) as evidenced by the pattern of relationships between items. Concurrent validity should be suggested by meaningful correlations with extant measures and the instrument should differentiate children diagnosed with and without autism. Ideally, the measure should also predict maintenance (or absence) of diagnosis as the child develops.

Finally, Level 2 screeners should discriminate children with autism from those with other developmental disorders (e.g., language disorders, intellectual disabilities, other neuro-developmental disabilities). This discrimination is typically evaluated using a signal detection theory (SDT) approach which offers several informative indices for establishing specific test cutoff scores to indicate likely presence of autism. For example, for any given cutoff on the test, sensitivity (S_E_) refers to the proportion of children among those known to have the condition who screen positive. Specificity (S_P_) refers to the proportion of children among those known *not* to have the condition who screen negative. In SDT parlance, S_E_ denotes the “hit” rate and 1–S_P_ indicates the “false alarm” rate. If the cutoff score is varied across the range of possible test scores, a range of operating points is obtained that summarizes the diagnostic performance of the test. Plotting these points produces a Receiver Operating Characteristics (ROC) curve which plots S_E_ values against 1–S_P_ values (i.e., hits against false alarms) across the range of possible cutoff values for the test. An area under the curve (AUC) statistic can then be used to assess the test's ROC performance (i.e., its discriminative performance). A test with an AUC = 0.5 is providing no predictive information whereas an AUC = 1.0 indicates a test that is providing perfect discrimination. In other words, when tests (or items) are contrasted via ROC analysis, the higher AUC denotes the more discriminating test (or item). Although the AUC is a commonly used statistic for evaluating discriminative performance, in our evaluation of Level 2 screeners we discuss potential problems that may be associated with its use. We emphasize that any assessment of the discriminative performance of an instrument must take into account considerations of sample size and statistical power if reliable conclusions are to be drawn about issues such as the most appropriate subset of prospective items to include in an instrument, the instrument's capacity to discriminate autism from other developmental conditions, and the comparative performance of different instruments.

## The Types of Behaviors Assessed by Screening Instruments

Although there is a to-be-expected overlap across instruments with respect to the behaviors addressed, there are differences between screeners. One reason for such differences is an historical one, with measures obviously shaped to some degree by the specific DSM (DSM-IV, DSM-IV-TR, DSM-5) (17–19) or the International Classification of Diseases [ICD-10, (20)] criteria operating when they were developed. Another reason relates to the length of the test, with more items providing opportunities to capture a wider range of behaviors or more subtle variations on particular types of behavior—objectives that must be traded off against that of having a screener that may be widely adopted because it is more efficient to administer. A third reason is apparent from a quick scan of the rather generic formal criteria. For example, items tapping a criterion for Autistic Disorder (AD) such as “Impaired social interaction, including … failure to reciprocate socially or emotionally” (DSM-IV-TR) or, for Autism Spectrum Disorder (ASD), “Sustained and widespread deficits in social communication and interaction, spanning the areas of social-emotional reciprocity” (DSM-5) can obviously be instantiated in various ways.

Perusal of the published studies describing content development for different tests reveals other influences. One factor is the test developer's clinical diagnostic experience and knowledge, which inform judgments about items that might best discriminate young children with autism from developmentally matched age peers (21). Other influences described by test developers are parents' retrospective reports of their child's behavior and behavioral observations coded from parents' home videos of their child (22), and research findings that highlight potential behavioral differences between children diagnosed with autism and typically developing (TD) children (22–24).

The reliance on these various influences as part of item development is not surprising if one attempts to interpret the diagnostic criteria in the context of the behavioral development of very young children. Consider, for example, DSM-5 criteria such as “Sustained and widespread deficits in social communication and interaction, spanning the areas of …developing and maintaining relationships” or “restricted and abnormally intense interests.” Their generic nature means that, although experienced clinicians might find it relatively easy to specify likely behavioral characteristics of children aged 4–5 years and older who don't meet specific criteria, their task will be more challenging when dealing with infants aged 9–24 months.

Rather than describing all behaviors from the numerous existing screeners, and showing how they link to DSM criteria operating when they were developed, we simply provide an overview of behaviors captured in four of the five Level 2 screeners described in more than one published evaluation and scrutinized in a later section—the Screening Tool for Autism in 2-Year Olds [STAT; (21)], the Baby and Infant Screen for Children with aUtIsm Traits–Part 1 [BISCUIT-Part 1; (25)], the Autism Detection in Early Childhood [ADEC; (22)], and an updated version of the Systematic Observation of Red Flags [SORF; (26)]—and indicate how they link to specific DSM-IV-R and DSM-5 criteria (see Table 1).

**Table 1:** Items from STAT (21), ADEC (8), SORF (26), and BISCUIT-Part 1 (24) linked to DSM-IV-TR and DSM-5 criteria.

	**Test items**			
**DSM version/** **criteria**	**STAT** **(11 items)**	**ADEC** **(16 items)**	**SORF (22 items)**	**BISCUIT—Part 1** **(54 items)**
**DSM-IV-TR (2000): Autistic disorder**				
A1-a Non-verbal behaviors		•Gaze monitoring •Reciprocity of smile •Use of gestures •Eye contact •Reaction to common sounds •Nestling •Anticipating social advances	•Warm expressions •Reduced facial expressions •Eye gaze to faces •Non-verbal communication	•Understand cues or gestures •Reads non-verbal cues •Too few or too many gestures •Gives subtle cues or gestures •Appropriate face expressions •Body posture and gestures •Use of facial expressions •Eye-to-eye gaze •Maintains eye contact •Displays range of appropriate facial expressions •Use of non-verbal communication
A1-b Peer relationships			Interest in objects over people	•Peer relationships •Make and keep friends •Social interactions with same age •Socializes with other children •Development of social relationships •Plays appropriately with others
A1-c Sharing enjoyment	•Play: turn taking •Requesting: snack •Requesting: bubbles •Directing attention: balloon •Directing attention: puppet •Directing attention: toys •Directing attention: noisemaker •Directing attention: rattle •Motor imitation: car •Motor imitation: drum •Motor imitation: hop dog	•Joint attention •Task switching •Imitation	•Sharing interests •Showing and pointing	•Motivated to please others •Shares interests with others
A1-c Social reciprocity		•Response to name	•Response to name •Using hand as tool •Reciprocal social play	•Interest in social games •Participates in games •Interest in other conversation •Understand appropriate jokes, figures of speech •Responds to others' cues •Make believe play •Responds to another's distress •Expects others to know their thoughts •Recognize emotions of others •Isolates self
A2-a Spoken language		•Delayed language	•Directed consonant sounds	•Use of language to communicate •Verbal communication •Communication skills •Language development
A2-b Conversation skills				•Use of language in conversations with others •Communicate effectively
A2-c Stereotyped language			•Repetitive speech	•Saying words/phrases repetitively
A2-d Play	•Play: doll play	•Functional play •Following verbal commands •Pretend play	•Clutches objects—or A3(d)	•Pretend play
A3-a Restricted interests			•Ritualized behavior •Excessive interest •Sticky attention	•Restricted interests •Limited number of interests •Restricted interests and activities •Curiosity with surroundings
A3-b Rituals/routines		•Ritualistic play and stereotyped behavior (& distress over change)	•Distress over change	•Upset if change in routine •Needs reassurance if things don't go to plan
A3-c Repetitive movements		•Ritualistic play and stereotyped behavior	•Repetitive movements	•Abnormal movements of whole body •Repetitive movements for no reason •Repetitive hand or arm movements
A3-d Preoccupation			•Repetitive use of objects •Fixation on object parts •Clutches objects—or a2(d)	•Fascination with spinning objects •Odd routines or rituals •Preoccupation with object parts
Not applicable			•Sensory aversion •Sensory interest	•Intellectual abilities •Age appropriate adaptive skills •Reaction to sounds/sights •Prefers food of certain texture/smell •Reactions to normal sounds •Reactions to normal lights
**DSM-5 (2013): Autism spectrum disorder**				
A1 Social-emotional reciprocity		•Response to name •Gaze monitoring •Joint attention •Following verbal commands •Reciprocity of smile •Task switching •Delayed language •Imitation	•Response to name •Showing and pointing •Interest in objects over people •Sharing interests •Reciprocal social play	•Interest in social games •Interest in other conversation •Shares interests with others •Isolates self •Use of language to communicate •Verbal communication •Communication skills •Language development •Use of language in conversation with others •Communicates effectively
A2 Non-verbal social behavior		•Eye contact •Nestling •Anticipating social advances	•Warm, joyful expressions •Reduced facial expressions •Eye gaze directed to faces •Using hand as a tool •Non-verbal communication •Fixation on object parts •Sticky attention	•Understand cues or gestures •Reads non-verbal cues •Motivated to please others •Responds to others' cues •Too few or too many gestures •Gives subtle cues or gestures •Body posture and gestures •Recognize emotions of others •Use of facial expressions •Eye-to-eye gaze •Maintains eye contact •Displays range of socially appropriate expressions •Use of non-verbal communication
A3 Developing relationships	•Play: turn taking •Play: doll play •Requesting: snack •Requesting: bubbles •Directing attention: balloon •Directing attention: puppet •Directing attention: toys •Directing attention: noisemaker •Directing attention: rattle •Motor imitation: car •Motor imitation: drum •Motor imitation: hop dog •Requesting: snack •Requesting: bubbles	•Functional play •Pretend play		•Peer relationships •Make and keep friends •Social interactions with same age •Socializes with other children •Development of social relationships •Plays appropriately with others •Participates in games •Understand appropriate jokes, figures of speech •Appropriate facial expressions •Make believe play •Responds to another's distress •Expects others to know their thoughts
B1 Repetitive motor behaviors, use of objects or speech		•Ritualistic play and stereotyped behavior	•Repetitive use of objects •Repetitive body movement •Clutches objects •Fixation on object parts	•Fascination with spinning objects •Restricted interests •Abnormal movements of whole body •Preoccupation with object parts •Repetitive movements for no reason •Repetitive arm or hand movements •Saying words/phrases repetitively
B2 Insistence on sameness, adherence to routines		•Ritualistic play and stereotyped behavior (& distress over change)	•Ritualized behavior •Distress over change	•Odd routines or rituals •Upset if change in routine •Needs reassurance if things don't go to plan
B3 Restricted and intense interest			•Excessive interest	•Limited number of interests •Restricted interests and activities
B4 Hyper- or hypo-sensitivity to sensory stimuli		•Response to everyday sounds	•Sensory aversion •Sensory interest	•Reaction to sounds and sights •Reactions to normal sounds •Prefers food of certain texture/smell •Curiosity with surroundings •Reactions to normal lights
Not applicable			•Directed consonant sounds	•Intellectual abilities •Age appropriate adaptive skills

*The STAT was developed in the era of the DSM-IV, prior to the DSM-IV-TR but, given it has been subjected to several evaluations between 2000 and 2020, its inclusion aids the demonstration of these links. Also, there is no attempt in this table to represent all criteria or the various contingencies that must be met (e.g., number of criteria met from each domain, when symptoms emerged, symptom severity) to meet the relevant DSM criteria*.

The key things to highlight from Table 1 are the following. First, most items from the four measures sampled in Table 1 illustrate (at least) one of the diagnostic criteria. Thus, instruments reflecting the DSM-IV-TR address impairments in social interactions (e.g., in non-verbal behaviors, peer relationships and sharing with others, social reciprocity) and communication (e.g., language delay, conversing, stereotyped language, play behavior), and repetitive behaviors and interests (e.g., intense restricted interests, rituals and repetitive behaviors, preoccupations). DSM-5 driven behavioral foci include deficits in social communication and interaction (e.g., social reciprocity, non-verbal social communication, relationships), and repetitive behaviors and interests (e.g., motor behaviors, insistence on sameness, rituals and intense interests, sensory sensitivities).

Second, different measures frame items differently and differ in their operationalization of the target behaviors and the way in which they access the information. Moreover, the importance placed on each behavior is reflected in the number of items probing that behavior. Nevertheless, a scan of Table 1 reveals a high degree of similarity between many items from the different tests that reference the same domain.

Third, the distribution of items across the different domains varies across measures, with the following quite pronounced differences. The ADEC has a higher proportion of its items aligning with DSM-IV-TR's non-verbal behavior domain (A1-a) and the DSM-5's social reciprocity domain (A1) than the other measures. The STAT is almost exclusively comprised of items compatible with DSM-IV-TR's sharing enjoyment (A1-c) domain. Unsurprisingly, items tapping repetitive and stereotyped behaviors and interests are more prominent in the post-DSM-5 measures (26), although they are certainly not absent from earlier measures (22, 24). Ultimately, of course, the number and proportion of items are not the key issues. Rather, the major considerations are whether the operationalization of items and data collection method ensure reliable measurement, the items discriminate as intended, and this discrimination spans the age range that early screeners are designed to target, and they have some predictive validity (i.e., the diagnosis is maintained).

## Level 1 Screeners

The focus for Level 1 screeners is large-scale screening of young children in the general population to identify potential concerns with the child's developmental trajectory. In the last two decades there has been a proliferation of Level 1 screeners, including (inter alia) well-documented instruments such as the Checklist of Autism in Toddlers [CHAT; (27)], the Checklist for Early Signs of Developmental Disorders [CESDD; (28)], the Early Screening of Autistic Traits Questionnaire [ESAT; (29)], The First Year Inventory [FYI; (30)], the Infant-Toddler Checklist [ITC; (31)], the modified Checklist for Autism in Toddlers [M-CHAT; (32)], and the Social Attention and Communication Study [SACS; (33)].

These measures differ in many respects. Here we provide just a few examples to highlight the extent of the differences. The number of items ranges from 4 on the pre-screening component of the ESAT to 63 on the FYI. Although Level 1 screener content reveals similar types of behaviors across instruments, the item list often differs depending on the age of the child being evaluated (28, 33). Items tapping similar aspects of behavior are operationalized with what appear to be varying degrees of precision, and coding might involve a simple yes/no or a rating on a Likert-type scale. For example, the CESDD requires observers reporting on the child's eye contact to tick the box “lack of eye contact” if the child's behavior is qualitatively different from their peers. In contrast, for the SACS, the observer is asked, “Has the child spontaneously made eye contact with you during the session? If not, interact with the child to elicit eye contact. Does he/she make eye contact with you?” and the observer is trained to identify and record whether the behavior is atypical (or typical). These different degrees of specification may or may not produce different profiles for the same behavior. For both instruments, observers completed a training workshop involving systematic instruction, video demonstrations of behaviors, etc. (28, 33).

Different instruments have also used different types of administrators, including physicians (e.g., ESAT pre-screener items), clinical psychologists (e.g., ESAT), child care workers (e.g., CESDD), maternal and child health-care center nurses (e.g., SACS), and parental reports (e.g., FYI, ITC). Thus, even with appropriate training, administrators are likely to differ with respect to clinical expertise, understanding of behavioral criteria, extent of exposure to the child, understanding of normative behavior patterns and so on. Moreover, the use of screening, and the likelihood of children receiving subsequent comprehensive diagnostic assessments, will also vary depending on whether the screening is embedded within a broader community health program (e.g., SACS) or is reliant on parents electing to volunteer.

Several broad observations have been made in evaluations of these instruments. First, the S_E_ of some instruments (e.g., CHAT, ESAT) appears to sub-optimal (27, 34). Second, some instruments (e.g., CESSD, CHAT, ESAT,) appear to generate relatively high rates of false positives (27, 28, 34). Third, where S_E_ and S_P_ appear to be more impressive (e.g., ITC), they may be overinclusive, not differentiating children with autism from those with language or other developmental delays (31). Also, the effectiveness of these instruments appears less impressive below 12 months (31) and is enhanced by repeated screening from 8 to 24 months (33).

Evaluation of Level 1 screeners is constrained by the fact that comparisons of different instruments' performance with the same samples have been scarce. Dereu et al. (35) reported comparisons of the CESDD (completed by child-care workers) with several parent completed measures—the ESAT, M-Chat (32), and the Social Communication Questionnaire [SCQ; (36)]—and concluded that they showed similar discriminative capacity. However, Dereu et al. noted a range of issues that clouded the interpretation of their findings, not the least being that the comparison sample contained children who had previously screened positive on the CESDD or had language delay.

The Level 1 screener literature suggests several broad messages. First, multiple screens in the first 2 years may improve both S_E_ and S_P_ by accommodating the variations in individuals' developmental trajectories. Second, a positive screen at a young age ideally should be followed up with either a subsequent screen to ascertain whether (a) the result may have been a false positive (followup with a Level 1 or 2 screener), and (b) the child is showing signs of autism or other developmental issues (followup with a Level 2 screener or comprehensive developmental assessment). Likewise, it would be ideal for negative screens to be followed up given the damage that may accrue when false negatives delay referrals for more comprehensive assessments. However, the issues associated with achieving such outcomes are, of course, complex and involve a variety of organizational and socio-political considerations—in addition to having discriminating test items. For example, the attainment of broad Level 1 screening is likely to be facilitated by the availability and accessibility of a measure that can be administered quite quickly (i.e., relatively few items), is not dependent on highly intensive training or specialist expertise, and can be scored quite easily. The likelihood of such a measure being widely adopted will depend on it being embedded within a community health assessment program that has strong and sustained governmental support and extremely broad outreach, such as that documented by Barbaro and Dissanayake (33). Realizing that objective may be more difficult than designing the measure.

Third, the Level 1 screening literature highlights the fact that, even if widespread adoption can be achieved, major obstacles to realizing the benefits that could flow from a positive screening outcome while the child is young are still likely to exist. For example, Barbaro and Dissanayake (33) found that only about 50% of the at-risk infants identified by the screening progressed to the comprehensive developmental unit associated with the research team (although the others may have followed other pathways). Issues associated with parental compliance with subsequent followups have been noted in some, although not all, other studies (28). Testimony to the importance of recognizing that there are likely to be important “system” variables constraining early detection is provided by Oosterling et al.'s (37) study. It revealed a lower mean age of autism diagnosis and higher proportions of diagnoses before 36 months in an experimental (compared with a control) region that integrated training of professionals in recognizing early signs, the use of a Level 1 screener accompanied by a specific referral procedure, and the availability of a multidisciplinary diagnostic team. The importance of these system variables was reinforced by an examination of the sustainability of that program. A cessation of funding and staffing support, that undermined staff training in recognizing early signs and using the referral protocol, saw the beneficial effects on early detection no longer sustained (38).

## Level 2 Screeners

The content of Level 2 screening instruments may often look similar to that of Level 1 screeners but the focus in instrument development and evaluation differs. Whereas, Level 1 screeners are probing in the general population for a potentially problematic developmental trajectory that may suggest the child is showing early signs of autism, and may indeed differentiate autism from other conditions, Level 2 screeners are designed to differentiate young children with ASD from those with other developmental disorders or concerns. Numerous Level 2 screeners have been described in the last two decades. Here we focus on five instruments that have been subjected to psychometric scrutiny involving either a large sample or several different samples, with the evaluation outcomes published in refereed journals. We review each of these instruments—the STAT, BISCUIT–Part 1, ADEC, SORF, and RITA-T. Other widely used instruments not specifically targeting very young children—for example, the Childhood Autism Rating Scale [CARS; (39, 40)]—are not considered in detail.^1^

### The Stat

The STAT was first described in an unpublished manual [see (21)] as a brief interactive measure that did not demand language comprehension and could be used by health-care workers and related professionals. A number of published evaluation studies have emanated from laboratories led by researchers from four different US institutions and from one Taiwanese university (21, 43–48).

The original target age range was 24–36 months, but it has subsequently been evaluated with children as young as 12 months. As shown in Table 1, it includes 12 social-communicative items administered in a play-based interaction session of about 20 min duration. The items deal with “negative” symptoms, that is, the absence of behaviors, with responses on each scored as pass/fail giving rise to both an area and a total score. Scoring criteria are set out in an instructional manual, with various options for training and certification in the use of the STAT available online.

Samples for the aforementioned studies were recruited from various sources including multidisciplinary evaluation centers, speech and hearing centers, research projects recruiting siblings of autistic individuals, children referred to an early intervention program from a community Level 1 screening program, etc. Total sample sizes (i.e., depending on the specific diagnostic categories captured, AD; Pervasive Developmental Disorder-Not Otherwise Specified (PDD-NOS); ASD, developmentally delayed (DD); language impaired, no ASD) varied from 33 to 382 (median = 104). Depending on the study, diagnoses against which these measures were validated were based on DSM-IV, DSM-IV-TR or DSM-5 criteria, sometimes using only a clinician diagnosis (21) but generally supplemented with standardized measures such as the ADOS, ADI-R, and other measures, with diagnosticians blind to STAT outcome in at least five of the published studies.

Whether the administrator was blind to referral reasons and diagnosis of the child in the studies cited above is sometimes clear (21, 43, 46, 48), but not always (45). Administrator training is usually carefully described, but inter-rater reliability data are presented meticulously in some papers (44, 46), quite vaguely (i.e., difficult to interpret) in some (43, 48) and not at all in others (45)—although reference to other published reliability data (46) or the meeting of online training criteria may be present (44, 47). Test-retest reliability data have been presented (47) but are seldom provided in subsequent studies using different samples and administrators.

Concurrent validity was demonstrated via the high agreement between the STAT's classification of a child as high or low risk for autism and their ADOS-G classification (46). The STAT's capacity to differentiate children with autism from other developmental disorders is illustrated by the S_E_ and S_P_ data reported in the seven evaluation studies cited above. Using the optimal STAT cut-off score identified within each of these seven studies, and the total score for the full scale, S_E_ ranged from 0.47 to 0.93 (median = 0.86) and S_P_ ranged from 0.69 to 0.86 (median = 0.80).

Several other findings from these studies should be noted. First, Stone et al. (47) reported substantially better S_P_ when they removed the youngest infants (12–13 months) from their 12–24-month sample. Second, Stone et al. (46) found that the STAT did not reliably classify children with a PDD-NOS diagnosis, suggesting that the detection of milder autism spectrum symptomatology may be less likely. Third, although the predictive validity of a positive screen at a very young age is a relevant consideration when evaluating these instruments, published data on this are (to our knowledge) limited. In Stone et al. (47), the average lag between screen and diagnosis was 15 months (*SD* = 5). Wu et al. (48) reported S_E_ and S_P_ of 0.86 and 0.71 (or 0.70 and 0.79, depending on the optimal cutoff adopted) given an average lag to diagnosis of 18.6 months (*SD* = 1.1). Thus, based on the screening information provided by the STAT, the likelihood that children will continue to meet diagnostic criteria throughout childhood, or the likely severity of the condition, cannot be estimated. Of course, if the child's positive screen were followed (as would be desirable) by an evaluation using a standardized diagnostic tool such as the ADOS or the ADI-R that, when administered beyond 2 years, suggests a stable diagnosis at least up to 9 years (49), the issue of the screener's predictive validity is less relevant at least for that child.

### The Biscuit–Part 1

The BISCUIT-Part 1 was designed by Matson et al. [(25), cited by (50)] as an informant interview with the child's primary caregiver, with the interview conducted within the framework of a state-funded early intervention program in Louisiana (USA). Several published evaluation studies have emerged from the Louisiana team (24, 50–53). Note, however, that each article examines different psychometric properties of the same instrument apparently drawn from the same referral base and thus could be viewed as components of a single psychometric evaluation. To our knowledge, evaluations by research teams at other sites have not yet emerged.

The age range of the sample targeted in the BISCUIT-Part 1 evaluations is 17–37 months. In most of the published evaluations, the number of items included was 62. Items captured all domains of the DSM-IV-TR and DSM-5 (Table 1) and were selected based on DSM-IV-TR and ICD-10 criteria, clinician observations and research findings. Items were read aloud to a child's parent or primary caregiver, each accompanied by age-appropriate qualifiers (not listed in the papers cited above). Test administration time is listed as approximately 20–30 min, depending on the child's characteristics. The response format involves a three-point Likert-type scale describing whether, on each item, the child compares to a TD peer as 0 (not different; no impairment), 1 (somewhat different; mild impairment), or 2 (very different; severe impairment). Assessors were credentialed in one of several relevant disciplines and received a day's training that included information on autism, the scales, practice, and so forth.

Children tested were in a state-funded service for families of infants with a developmental delay or another condition likely to produce some delay. Sample sizes varied depending on the focus of the study (e.g., depending on the specific diagnostic categories included) and, compared to other screener studies, were typically much larger, ranging from several hundred to over 3,000. In some published studies the sample was obviously the same; in others the sample is described as a rolling sample, with children being progressively added to the cohort as time passed. Thus, some children appear in multiple studies, though the proportions doing so are unclear.

All diagnoses of AD or PDD-NOS were made by an experienced clinical psychologist in the field who was blind to BISCUIT scores. The diagnoses were based on clinical judgment using some combination of the DSM-IV-TR algorithm for AD, DSM-IV-TR descriptors for PDD-NOS, M-CHAT scores, and developmental profiles on the Battelle Developmental Inventory-Second Edition (54). It is unclear if this combination was available for each diagnosis and, if not, what proportion of diagnoses depended on each indicator. Nevertheless, in two of the papers cited above where another psychologist also provided a diagnosis, inter-rater agreement for diagnoses based on subsets of cases (*N* = 97 and *N* = 203) was impressively high, *k* = 0.98 and agreement = 98.7% (24, 52).

Reliability data for the BISCUIT are scarce. When examining the original list of items, Matson et al. (50) report item-total correlations and inter-item correlations suggestive of a coherent item set. Also reported is coefficient alpha—now heavily criticized as an index of reliability (55, 56). Moreover, at least in the “BISCUIT” papers cited above, neither test-retest nor inter-rater reliability data for the BISCUIT (as distinct from diagnosis) are reported.

Validity data for the measure were presented more thoroughly. Examination of the factor structure highlighted three factors—deficits in socialization, restricted interests and repetitive behaviors and communication—that align with characterizations of autism symptomatology (24). Convergent validity was suggested by robust correlations with the M-CHAT and the socialization skills domain of a standardized index of developmental functioning (51). The optimal cut-off score suggested by the authors for differentiating autism from PDD-NOS gave rise to S_E_ and S_P_ of 0.84 and 0.83, respectively (52), with the corresponding values for PDD-NOS vs. no diagnosis being 0.85 and 0.86. More recently, with a larger sample, S_E_ and S_P_ were examined for different age levels. For children aged 17–23 months, the optimal cut-off scores for differentiating autism from PDD-NOS produced S_E_ and S_P_ values of 0.80 and 0.81; the corresponding values for differentiating PDD-NOS and non-ASD related atypical development were 0.93 and 0.76 (53). No predictive validity data have been reported.

### The ADEC

The ADEC was first described in a published test manual (22) (Note that two of the authors of the current paper were involved in the ADEC's development). One objective was to detect pre-verbal behaviors that predict the development (or emergence) of AD in children under 3 years. The second was to operationalize these behaviors so precisely that clinicians with minimal expertise in autism could readily learn to administer the instrument within 10–15 min. Subsequent published evaluation studies have been led by researchers from four different Australian and US institutions (15, 57–62) with a further study conducted in Mexico using a Spanish translation (63).

The ADEC includes 16 items from the domains of disturbances in interacting with others and objects, stereotyped and repetitive movements, and bizarre responses to environmental stimuli (Table 1). Items are administered in a play-like interaction session involving the child, the tester and a parent or caregiver. Item development was guided by (a) core deficits suggested by the broad literature, (b) retrospective parental reports (8), and (c) analysis of home videos of infants subsequently identified with autistic disorder, developmental or language delay, or TD (64). The operationalization of these behaviors, scoring criteria for each item (0 = appropriate behavior, 1 = somewhat inappropriate, 2 = clearly inappropriate behavior) and lists of examples and non-examples are precisely specified in the manual, together with specific administration instructions. Thus, for example, a score of 1 might be received by a child who did not perform the behavior when required but at some other stage performed it spontaneously, or only displayed the behavior on some of the required attempts to elicit the behavior.

Extensive piloting of the instrument, often involving children older than 3 years (e.g., *M* ≈ 40 months), with a confirmed diagnosis of AD, other disability or TD, is described in the published manual and indicated impressive inter-rater and test-retest reliability, concurrent validity with the CARS, CHAT, and ADI-R, and promising diagnostic discrimination, S_E_ and S_P_. Other promising reliability and validity pilot data were provided in a study using a Spanish translation (63), but there were too few children under 3 years to allow any decisive conclusions regarding the instrument's merits for use in the target population.

Samples in the published ADEC studies cited above were recruited from state and privately funded centers for children referred with various developmental concerns, a university-based research center, a child development clinic of a large pediatric clinic hospital, and via general advertising. Total sample sizes (i.e., depending on the specific diagnostic categories captured, AD, PDD-NOS, ODD, TD) varied from 53 to 270 (median = 112).

The key studies we discuss in this section are Nah et al. (61), Hedley et al. (59) and Nah et al. (60), with the latter's sample overlapping with Nah et al. (61). In Nah et al. (61), children's ages ranged from 12 to 36 months, with mean ages in months for the various sub-samples of 29.4 (AD), 28.2 (PDD-NOS) 24.1 [other Developmental Disorder (DD), and 23.5 (TD). In Nah et al. (60), children's ages ranged from 12 to 36 months, with mean ages in months for the various sub-samples of 29.4 (AD), 28.2 (PDD-NOS) 24.1 (DD), and 23.5 (TD). Hedley et al.'s (59) children ranged from 14.3 to 36.9 months (*M* = 28.7, *SD* = 5.4); autistic children ranged from 19.2 to 36.9 months and the DD group from 15.9 to 36.8 months. A best estimate clinical (BEC) diagnosis was made by an experienced clinician who was blind to ADEC outcomes and used information from a variety of sources including the ADOS and ADI-R—but not the ADOS Toddler (5) or the ADI-R Toddler (65) designed specifically for children below 24 months]. Hedley et al.'s (59) diagnoses were based on DSM-5 criteria. The two earlier studies used DSM-IV criteria, with independent confirmatory diagnoses reported for 77.5% of participants with autism or DD diagnoses. The remaining ADEC studies cited above (15, 62) are discussed in a later section dealing with a brief version of the ADEC.

The test manual (22) reported impressive inter-rater agreement for a subset of the sample, with similar values of 0.98 (61) and 0.95 (59) reported subsequently. Test-retest reliability, after an average interval of 54.5 days, was 0.72 (61), but has not been reported in other studies.

The validity analyses reported in these studies yielded relatively consistent patterns. Nah et al. (61) conducted a factor analytic examination of the ADEC which indicated a one-factor (social communication) solution. In the same study, concurrent validity was indicated by robust correlations between ADEC total score and the sub-scales of the ADOS and ADI-R (except for the restricted and repetitive behaviors sub-scale of the latter). Hedley et al. (59) also reported strong correlations with ADOS-2 sub-scales and with the CARS. Both studies found that the ADEC predicted diagnostic status, whether it was DSM-IV or DSM-5. Nah et al. (61) also demonstrated diagnostic validity with ADEC total scores discriminating AD, PDD-NOS and DD, with this pattern prevailing even after controlling for non-verbal IQ and adaptive behavior, and in a subgroup aged 24 months or younger. In a similar vein, Hedley et al. (59), confronted with a sample of autistic children of a lower developmental level than both the DD and no diagnosis comparison groups, matched infants on age, adaptive behavior and developmental quotient and reported that the ADEC still discriminated the autistic children from those with other developmental disabilities. Nevertheless, as previously noted (61), given the relatively low developmental levels of their AD sample, further work is needed to clarify the discriminative capacity of the ADEC when used with more developmentally advanced autistic children.

For both the full AD and DD samples, and for AD and DD samples matched on age, non-verbal IQ and adaptive behavior, Nah et al. (61) examined S_E_ and S_P_ for the following contrasts: AD disorder vs. DD, and AD vs. DD + TD. For the full samples, using the optimal, and the manual recommended, total score cutoffs, S_E_ and S_P_ for these two contrasts were 1.0 and 0.77, and 1.0 and 0.89, respectively. For the matched samples, the corresponding values the were 1.0 and 0.74, and 1.0 and 0.90, respectively. In other words, S_P_ was lower when TD children were excluded from contrasts. In Hedley et al. (59), whose sample included no TD children—but, unlike Nah et al. (61), were selected using DSM-5 criteria—S_E_ and S_P_ at the corresponding cutoff were 0.93 and 0.64.

It is interesting that the accuracy of screening clinicians' ADEC judgments about the presence or absence of autism was related to their judgmental confidence. Clinicians with experience in screening rated their confidence in their screening test judgment on a scale ranging from 0% (not confident at all) to 100% (absolutely certain) (58). Confidence was predictive of a subsequent accurate diagnosis only when it was in the range of 70–100%. Lower confidence ratings predicted accuracy of diagnosis at no better than chance levels. Subsequent research with samples sufficiently large to enable a more precise examination of the confidence-accuracy relationship may indicate how best to exploit experienced assessors' confidence judgments about the ADEC screen result when planning subsequent followup assessments.

Finally, predictive validity data are available from two studies, although in both cases the sample sizes are very small. Nah et al. (60) presented ADEC, and CARS, screening data for 55 children, 67% of whom were participants in Nah et al. (61). Comprehensive diagnostic assessments were conducted at the initial and two followup assessments, with assessors blind to the child's outcomes on the ADEC and CARS. The initial assessment occurred at 19–42 months (*M* = 33.5, *SD* = 5.6), with the sample comprising 51 who received a BEC DSM-IV-TR AD diagnosis, 2 with PDD-NOS and only 2 with non-autism spectrum disorders. Followups occurred 2 and 6 years later, though the latter followup was only available for 22 children. Both measures predicted an autism diagnosis at 2 years but not at 6 years, although combining individuals diagnosed with AD and PDD-NOS resulted in most children retaining their diagnosis after 6 years. The ADEC was also able to predict symptom severity at 6 years. Dix et al. (57) also reported ADEC data for a small sample of 53 children (*M* = 32.2 months, *SD* = 8.4) referred for developmental concerns or autism risk and subsequently followed up 49–97 months later. Diagnoses were made jointly by an appropriate multidisciplinary team using DSM-IV-TR criteria and including the ADOS and ADI-R, with age at diagnosis ranging from 22 to 65 months (*M* = 41.2, *SD* = 9.2). At the followup, 60% had received an ASD diagnosis and 36% had developmental delays. S_E_ and S_P_ were 0.88 and 0.62, respectively.

### The SORF

A published version of the SORF appeared in Wetherby et al., (66), with teams (led by Dow, a US researcher) subsequently reporting formal evaluations of a modified form of the SORF as a Level 2 screener (26, 67). The original version comprised 29 items based on DSM-IV criteria, with item content later modified to include 22 items based on DSM-5 criteria. The two evaluation studies (26, 67) studies examined the screening performance of the SORF with infants ranging in age from 16 to 24 months when applied in conjunction with a video-recorded administration of Wetherby and Prizant's Communication and Symbolic Behavior Scales (68), and coded by individuals without expertise in autism. Note that the first author of the Dow et al. evaluation studies (26, 67) has indicated that the two samples overlapped (exact degree not specified), with some children being coded for the SORF based on both clinic observations (26) and home observations (67). The degree of overlap has potentially implications for the likely generality of the findings.

The social communication and restricted and repetitive behavior domains of the DSM-5 are each represented by 11 items. The children were recorded interacting with their parents in a range of activities in their home environment for 1 hour. Undergraduate coders, blind to diagnosis, received training on diagnostic features of autism and the coding system, then watched a 20 min (26) or 1 h (67) recording of the interview. Using a 0–3 response scale (0 indicated absence of relevant concern, 3 indicated the greatest level of severity or concern), they rated the presence/absence of behaviors referred to by the items—see Wetherby et al.'s (66) Appendix A for detailed descriptions—that are atypical/typical of TD children. Inter-rater reliability was indicated by intraclass correlation generalizability coefficients for the total composite score for the best performing 17 items (26) and 6 items (67) of 0.86 and 0.75, respectively. No test-retest reliability data were reported in these papers.

Samples were recruited to a longitudinal, prospective study of autism and communication disorders. The children were referred via primary care screening and met the criteria that the SORF and a diagnostic assessment had been completed between 16 and 24 months. In Dow et al. (26), children's mean ages were 20.8 months for each of the autistic, DD and TD sub-samples; the corresponding values in Dow et al. (67) were 20.7, 20.3 and 20.4 months, respectively. The autistic and DD children did not differ significantly on the non-verbal development quotient measure used; importantly, the sub-samples performed at a higher developmental level than those reported using the same developmental scales in the STAT and ADEC evaluations of Stone et al. (46), Nah et al. (61), and Hedley et al. (59). BEC diagnoses in both Dow et al. (26, 67) studies were based on a combination of sources including the ADOS-T, developmental and adaptive behavior scales, and the video-recorded observation session.

Outcomes for the validity analyses were similar in the two studies. Both studies reported diagnostic validity with the respective composite (and other summary) scores discriminating the autistic and non-autistic children. Using the optimal composite scores, S_E_ and S_P_ for the contrasts of autistic and non-autistic children were 0.80 and 0.78 (26) and 0.77 and 0.72 (67), respectively. No predictive validity data were provided.

### The RITA-T

Our discussion of the RITA-T is much briefer than that of the preceding instruments because it needs to be viewed as being at an early stage of evaluation. The two main data-based publications are a small sample study reported by US-based researchers, Choueiri and Wagner (23), and a larger-sample study recently described by Canada-based researchers, Lemay et al. (69). The measure involves 9 semi-structured and play-based activities that are administered and scored in a 10-minute session, with very modest administrator training requirements. The samples ranged in age from 18 to 36 months.

We have included this measure because of two interesting features. First, the activities, or items (23), which cluster exclusively under domains A1 and A of the DSM-IV-TR and DSM-5, respectively, are operationalized a little differently to items in other measures. Second, S_E_ and S_P_ at the optimum score cutoffs identified are promising, reported as 1.00 and 0.84 (23) and 0.97 and 0.71 (69). Although these features of the studies should pique researchers' interest, there are several reasons why the findings should be regarded as preliminary.

First, in neither study was a measure such as the ADOS administered to all at-risk children; nor were diagnoses independently confirmed. Second, the inter-rater reliability protocol lacks clarity and no test-retest data were reported. Third, in the large-sample study (Lemay et al.), all children had been referred to the clinic with ASD concerns and diagnosing clinicians could have accessed the RITA-T administrators' clinical observations. Fourth, Choueiri and Wagner's conclusion that their ASD and DD sub-samples were of comparable developmental levels is not justified, despite their reporting non-significant differences between the samples. Those contrasts were clearly under-powered, and the descriptive statistics strongly suggest that developmental level should have been controlled in analyses. Moreover, the samples were too small to match on developmental level as was done, for example, by Nah et al. (61). Further, in Lemay et al., no data are provided on developmental level for their ASD and non-ASD groups.

### Level 2 Screeners: A Summary

What should we conclude regarding the utility of these screeners? Some researchers—perhaps all—not surprisingly, appear to favor their own measure. For example, Dow et al. (26, 67), while acknowledging important unanswered questions about the SORF, appear to be leaning toward the superiority of the SORF on the (reasonable) basis that the items reflect DSM-5 criteria and their sample is younger, a little bit larger, and of a higher developmental level than those in some of the other papers they cite. We, however, are unsure because the various evaluation studies differ in so many potentially important respects: sourcing of the samples (e.g., primary health care referrals vs. clinical samples) and the likely prevalence and severity of individuals at risk; sample sizes; ages and developmental levels of individuals sampled; rigor and reliability of diagnoses; whether the individuals were classified according to DSM-IV-TR or DSM-5 criteria; precision of operationalization of target behaviors; quality of rater training; reliability of the raters and stability of those ratings, and so on. The instruments also differ in terms of the very important criterion of whether they have been subjected to any evaluations by researchers beyond the laboratory or clinic of the test developers, let alone in different countries and cultures. And although the S_E_ and S_P_ indices suggest variations in the discriminative performance of the instruments, who knows which of the many variables mentioned above contribute to those variations. Maybe these apparent performance differences reflect differences in the overall package of items or the testing methodology—but perhaps they just reflect one or more of the other factors listed above. The picture is so complicated that, although all the measures offer potential, we are not prepared to make an unequivocal case for the superiority of any of them in terms of discriminative performance.

Each measure is deficient in one or more of the following respects. Although independent evaluations (i.e., from other labs or clinics) have appeared in refereed journals for the STAT, ADEC, and RITA-T, this is not yet the case (to our knowledge) for the SORF or the BISCUIT. Large sample sizes—obviously an issue in research with autistic individuals—at all ages within the range of interest and encompassing different developmental levels is an issue for all instruments (except the BISCUIT). Demonstrations of inter-rater reliability and the stability of the screening measure—at least in the refereed publications—are not always apparent, or the protocols are vaguely described; nor is the independence of screening outcomes and diagnoses always unambiguous. And, even though the published papers on the STAT, ADEC, and SORF report reliability data systematically, we question whether sufficient attention is being paid to reliability issues. The various dimensions of test reliability not only have implications for the discriminative performance of a test in any individual study but, given the nature of the decisions made on the basis of these screening instrument outcomes in individual cases, they are also potentially of great significance for the young children and their families. Thus, a reasonable question to ask is whether those reliability levels that are cited as acceptable, or even impressive, for research purposes should be considered adequate when critical decisions are being made that are likely to shape the lives of an individual child and their parents. Complicating this issue specifically with respect to test-retest reliability is the heterogeneity of the condition and the way in which it unfolds. Consequently, although test outcomes should be stable over short test-retest intervals, fluctuations might be expected after longer intervals, thereby emphasizing the importance of multiple assessments. Finally, the relatively small sample sizes in all studies make it difficult to determine whether the (likely heterogenous) “other developmental disorder” sub-samples that are such an important part of Level 2 evaluations effectively represent the different conditions whose symptoms are perhaps most likely to be confused with ASD.

Taken together, such concerns highlight that missing from the field is any systematic comparison of these measures under consistent conditions, with substantial sample sizes, and adequately capturing the diversity of other potentially confusable developmental disorders. One sensible way to achieve such objectives would, therefore, be cooperation between researchers (something that is becoming much more prevalent in many areas of science). Of course, even if all those conditions were met and one measure appeared to outshine the others, it does not mean it should necessarily be the go-to measure. As we noted earlier when discussing Level 1 screeners, these instruments are used in diverse organizational systems to guide delivery of assessment and intervention services. Thus, for example, the availability of comprehensive followup assessment services and the known effectiveness of particular intervention programs are likely to influence judgments about the specific age and developmental levels that a screener should be targeting. Moreover, in some contexts an elaborate, time-consuming screening process conducted by professionals may be readily accommodated; in others the way forward may be via a speedier process routinely administered by professionals with minimal training. Or, the particular assessment and intervention context may well shape how S_E_ and S_P_ are prioritized and, hence, whether clinicians rely upon the optimum score cut-offs identified in the published evaluations or favor a different cut-off that increases S_E_ at the expense of S_P_ (or vice versa).

The importance of these system variable considerations is suggested by the difficult to resist observation that many screener projects look like the work of an enterprising clinic, laboratory or individual, with only the STAT and the ADEC (among Level 2 screeners) thus far revealing evidence of long term and significant cross-institutional followup. Access to the requisite samples is obviously one limiting factor. And in most research areas, turning scientific research into specific and influential practical outcomes can be extremely difficult. Organizational imperatives will likely vary from region to region, state to state, and country to country. The ability to negotiate a way through those imperatives may prove to be far trickier, and ultimately much more significant, than any subtle differences in screener efficacy. Consequently, we need to identify and understand potential organizational and cultural constraints and to be able to make clear economic and socio-political cases for the advantages of screening. Inevitably this will involve providing precise information on costs and benefits—both economic and social—for the children, their progress to adolescence and adulthood, as well as for their families and service providers.

In addition to the organizational considerations that are likely to shape decisions about the selection of an appropriate screener, its implementation, the interpretation of the test outcomes(s), and subsequent decisions about referral for further assessment and intervention, there are some important practical issues that we review in the next section.

## Some Practical Issues

Many of the practical barriers to the adoption of screeners and their effective use have already been canvassed widely in the literature, including a comprehensive and concise overview (16). Some of these barriers have already been mentioned in our concluding remarks on Level 1 screeners and are equally apposite here. There are, however, three issues that we wish to emphasize, two of which have been covered in some form elsewhere in the literature or briefly mentioned in earlier sections.

The first issue is that a screen (or diagnosis)—negative or positive—at a very young age should not be seen as a single-point event given developmental trajectories may vary in unpredictable ways. Although the stability of diagnoses at around 18–24 months appears to be high across different sample types, an early negative screen does not guarantee that autism symptoms will not emerge at some later date. One study, for example, with a sample of children at familial risk, reported high diagnostic stability at 36 months for children detected at 18 and 24 months and, yet, nearly half of the sample were not identified at 24 months but were diagnosed at 36 months (70). In a similar vein, longitudinal data from later-born siblings of children with autism revealed that, despite multiple negative assessments in the preschool years, some met criteria for autism when reassessed in the 5–9-year age range (10). The authors suggested that, in some children, autism symptoms may continue to evolve after only showing quite subtle signs at younger ages. Others have reported that some children who showed regression (loss of skills) as they approached 24 months had previously appeared to be developing typically (71). Similarly, a false positive for autism on a single early screening test should not then be regarded as a guarantee of a typical developmental trajectory, as researchers have demonstrated that significant proportions of such children are likely to be at risk for various other developmental disorders that should be the focus of systematic observation and assessment (72, 73). Of course, it is also possible that an individual's trajectory may change if they are screened and subsequently exposed to some systematic intervention program that perhaps moderates their ASD symptomatology or influences the manifestations of other (potentially confusable) developmental conditions. In sum, as we noted when discussing Level 1 screeners, ongoing monitoring and assessment of children who appear at risk has the potential to aid the early detection of symptomatology of autism and other developmental disabilities. Moreover, a focus of such monitoring should be on younger siblings of children already diagnosed with autism given the heightened level of risk (16).

The second issue is one that has assumed prominence given the spread of Covid-19. The widespread distribution of the virus and its devastating consequences have had significant implications for those seeking and delivering health care services. First, with communities in isolation for extended periods it seems possible that the likelihood that parents would seek professional advice when they suspected developmental issues with their children would have fallen. Second, the accessibility of face-to-face services likely diminished. Under such conditions, telehealth services become critically important, just as they are for people who live in remote regions.

Researchers have taken up the challenge in the areas of assessment (74) and intervention (75), although it is still “early days” in terms of delivering assessment using this approach. For some Level 2 screeners the adaptations required will be less demanding than for others. For example, the administration of the SORF (67) involved video recording of parents interacting with their child in a variety of activities during a 1-hour session, with coding done by research assistants using the video. For measures that involve a more structured interaction between assessor or parent child (e.g., the ADEC), the use of telehealth assessments that may by necessity need to be parent-led will need to be evaluated to ensure fidelity of administration and reliability of the assessment. Nevertheless, at face value it would be surprising if these objectives were not achievable.

The third issue relates to parameters of the instrument that might lend it to being readily integrated into existing assessment frameworks. Within many primary care and organizational contexts, the likelihood of Level 2 screeners being used when appropriate may be enhanced by the availability of a measure that can be administered and scored in a timely manner—that is, few items and a brief administration time—and is not dependent on highly intensive training or specialist expertise. These practical considerations may also be particularly relevant in resource scarce contexts and where few professionals have expertise with comprehensive diagnostic techniques (e.g., developing countries).

Considerations of this nature have spawned interest in the development of short forms of Level 2 screeners. We consider these short forms here in some detail for two reasons. First, they obviously meet the practical efficiency criterion for instrument evaluation that we outlined earlier. Second, our examination of their performance will highlight an area of concern associated with evaluating the discriminative performance of different measures, a concern that has wider implications for the ongoing evaluations of Level 2 screeners and their item content.

### Short Forms

Researchers have published evaluations of short forms of two of the Level 2 screeners discussed in the previous sections: the ADEC and the SORF. In each case the evaluations have involved examining the performance of the best performing subset of the full item set in discriminating children with autism from other developmental concerns. In other words, the children were originally assessed using the full measure. The data for the best performing 6 items of the SORF have already been discussed (67). We do not discuss it further here because scoring the 6 items still involved a 1-hour video observation session. It will be interesting to see how that measure performs if based on a much shorter observation sample that reduces overall administration time significantly. In contrast, administration time for the full ADEC is only 10–15 min, with the short form discussed below taking less time.

Two studies have explored possible short forms of the ADEC (15, 62). Nah et al. (15) had 270 children aged 12–36 months, 197 of whom were part of Nah et al.'s (61) sample: based on BEC DSM-5 diagnoses, there were 106 (ASD), 86 (non-TD), and 78 (TD), with mean ages of 28.7, 23.1 and 23.5 months, respectively. Inter-rater reliability between two trained and experienced clinicians blind to the other's diagnosis was high (*k* = 0.96).

Nah et al. (15) specifically targeted a five-item version of the ADEC that could be administered and scored within 5 min. Those items—response to name, reciprocity of smile, joint attention and social referencing, following verbal commands, and use of gestures—were the items that yielded the highest AUC when comparing the autism and non-TD group, and together they formed the brief ADEC, or BADEC. The optimal cut off score yielded S_E_ =.81 and S_P_ =.78 (for ASD vs. non-TD) and 0.91 and 0.81 (for ASD vs. non-TD + TD). For the full ADEC, the corresponding S_E_ and S_P_ values for the former contrast were 0.87 and 0.84. The BADEC also demonstrated concurrent validity with the ADOS and ADI-R, and diagnostic validity with non-verbal developmental quotient controlled. Although the latter finding suggests the BADEC is detecting autism and not simply low cognitive functioning, we again emphasize that more work is needed to address the performance of the BADEC with autistic children who are at more advanced developmental levels.

Nevill et al. (62) replicated Nah et al. (15) using a US clinical sample [previously described in Hedley et al. (59)]. The sample included 110 children aged 14–36 months (*M* = 28.8), 49 with a confirmed ASD diagnosis and 61 without ASD. As in Nah et al. (15), diagnostic validity with non-verbal developmental quotient controlled was reported for total score on the best performing 5 ADEC items, and strong correlations with the ADOS and CARSs emerged. The best performing five item cutoff score (albeit with a more stringent cutoff than in Nah et al.) that optimized the S_E_-S_P_ balance produced S_E_ and S_P_ indices of 0.77 and 0.86, respectively.

Nevill et al.'s best performing five items were not identical to those found by Nah et al. (15). To identify the best performing five items, each research team conducted ROC analyses and then simply selected the items that produced the highest AUC values (i.e., best discriminated ASD from non-ASD). Three items were common to both studies: following verbal commands, response to name and reciprocity of smile. For Nah et al., the remaining two items were use of gestures and joint attention and social referencing; for Nevill et al. they were gaze monitoring and task switching. Nevill et al. suggested the different outcomes perhaps reflected sample characteristics and that the careful approach might be to use all seven items.

These two studies suggest that a short form of the ADEC—whether it be a five- or seven-item version—might constitute a useful practical addition to the range of Level 2 screeners because of ease of administration and coding. Before advocating a specific short form version based on these two studies, however, we speculate on a critical issue that those item comparisons have brought to the fore—the use of AUC to evaluate test performance—one that has significant implications for all evaluations of Level 2 screeners and their item content.

#### Using AUC to Evaluate Discriminative Performance

As is common practice, in the two short form studies discussed above the discriminative performance of the various ADEC items making up the short forms was evaluated by calculating area under the curve (AUC). For both of these short forms (15, 62)—as has been the case in numerous other evaluations of the diagnostic merits of screener items and tests—AUC values were contrasted without the use of any inferential test to determine whether the AUC differences are meaningful.

We suggest that interpretation of AUC differences when assessing the discriminative performance of different items or, indeed, different tests needs to be on a firmer footing than that provided by an eye-balling of AUC values (76), as was the case in the two short form studies discussed above (15, 62). Whether inferential testing reveals meaningful AUC differences will obviously be dependent on sample sizes. Given that in much of the Level 2 screening research sample sizes are modest, substantial AUC differences will be required to detect meaningful differences between items or measures. However, one cannot simply infer based on sample size whether two AUCs are likely to differ significantly because (a) the correlation between items will also affect the significance of the difference between two paired AUCs and (b) the correlation between items will not necessarily be stable.

In sum, truly meaningful AUC comparisons will be possible in contexts where the samples are sufficiently robust for the detection of reliable AUC differences. Therefore, in the absence of some data simulations that vary sample sizes and inter-item correlations while maintaining the AUCs reported by Nah et al. and Nevill et al., we are not prepared to arbitrate on which of the two subsets of ADEC items might provide better discriminative performance. We also emphasize that these considerations are relevant more generally for comparative evaluations of the discriminative performance of individual items and tests.

## Advancing the Field

In this section, we highlight four issues that we believe are priorities for future research, some of which have already been foreshadowed. The most important objective should be to devise and execute an approach to collecting sufficiently large sample sizes, originating from diverse referral sources, to allow realistic appraisals of Level 2 item and instrument discriminative capacity right across the 12–36 month age range. Only then will researchers be able to compare with authority the contributions of different items, the performance of the instruments as a whole, the capacity of observers to reliably code behaviors that are operationalized in different ways, and the stability or test-retest reliability of different items and measures. Realizing this objective will be a complex task and likely will only be achievable with multi-site approaches to study design and data collection. Such an approach may provide a number of benefits. It would help overcome a major limitation of existing research: namely, the limited evaluation of measures beyond the confines of the clinics and labs of the developing clinicians and the samples they are able to access. In so doing it might help the field progress beyond its current dependence on clinical judgments based on poorly operationalized and ever-changing DSM criteria, relying instead on standardized and meticulously operationalized instruments that provide a universal protocol for the diagnosis of autism.

A collective large-sample approach might also expedite dealing with reliability issues about which we can, at present, only speculate. It seems trite to say that it is highly desirable for items to be operationalized in ways that permit (with standardized training) perfectly consistent administration, interpretation and coding, but we cannot help but think that a focus on these issues that goes beyond the reliability criteria generally considered acceptable for research purposes might reap rewards. We wonder, for example, if we were to take multiple random draws of five or six items—with each item operationalized precisely and able to be scored with very high reliability—from a larger pool of well-targeted items (as in the short forms discussed) and administer them to very large samples, whether we might discover that the specific item content turned out to be less important than the precision of measurement.

A second objective should be to continue the search for item content, or behavior, that is discriminating and/or particularly so at certain age levels. Different lines of research have focused on non-social behavioral indicators that may be discernible prior to the emergence of social communicative deficits. A recent comprehensive review of such research (77) examined observable non-social behaviors in domains such as attention, visual processing, motor development, and repetitive and stereotyped behavior, exploring possible differences between younger siblings of children with ASD vs. siblings of TD children. Although the findings within many domains were non-significant or mixed, in some domains the balance of evidence suggested differences by around 12 months between siblings at elevated risk for ASD when the outcome was either later emergence of ASD or typical development. Impairments in the former group were detected in domains such as disengagement of attention (from a stimulus already engaged), motor development, repetitive interests and behaviors, and atypical sensory behaviors that, to date, have generally received less attention in screeners.

Examination of item content of current Level 2 screeners shows that they generally include items that tap into the just mentioned behavioral dimensions in some form, although more so if developed based on DSM-5. The challenge for the development of screeners, of course, is having items that are amenable to administration in a relatively brief testing session with the child and/or parent. The sometimes quite sophisticated paradigms that researchers have developed to probe specific processes and behaviors in different non-social domains may—given their measurement precision, their use of repeated trials and so on—be able to reliably detect fine behavioral impairments that are predictive of emergent ASD far better than their more “clumsy” equivalents that are typically seen in screener items. Although such paradigms can provide excellent research insights and may be able to be adapted for subsequent comprehensive assessments, translating them into a screener item that in one or two trials will produce a stable and discriminating measure will often be more difficult. Consequently, at least when it comes to probing some behaviors, it is likely that a considerable research effort will be required to bridge the gap required to translate a sophisticated lab based technique or measure into easily administered clinical test items.

A third focus for future research that has enormous implications for screener development should be trying to unravel the developmental trajectories of ASD in young children. We have highlighted research illustrating the problems that can arise when single-point screening of children deemed at risk is the norm. Although screening at several points in the early years is likely to reduce such problems, decisions about when to screen, screener content, or when to refer for more thorough assessment or intervention would be much better informed if we had a comprehensive understanding of how and when the condition may unfold. There exists a substantial body of research from various disciplinary areas that has contributed to our current understanding of developmental patterns (78). A challenge for clinicians interested in refining screeners will be to keep abreast of that literature, a literature that almost certainly will continue to burgeon, and to integrate the diversity of findings to achieve a coherent understanding of the development paths of ASD. Most, if not all, of the above suggestions apply equally to the refinement of Level 1 screeners.

Finally, at the risk of being way too repetitive, we conclude with a reminder that screening, and all the associated decisions about the measures used, any subsequent assessment, intervention and so on, occur within complex organizational systems and structures. Understanding the constraints those systems impose and how to shape them must be an ongoing focus.

## Author Contributions

NB drafted the manuscript in consultation with RY. CL drafted the section on AUC. RY and CL commented on the manuscript. All authors contributed to the article and approved the submitted version.

## Conflict of Interest

Flinders University receives royalties from sales of the ADEC.

## Footnotes

^1^Note that although the CARS has been criticized for over diagnosing infants (41), others argue that it may be useful for distinguishing autism from other developmental disorders as early as two years [e.g., (42)].
